# Ordinal measurement invariance of three retained GAEU-1 dimensions in Chilean and Ecuadorian university samples

**DOI:** 10.3389/fpsyg.2026.1990515

**Published:** 2026-10-15

**Authors:** Xinglong Pang, Lin Zhou, Jin-Ho Cho

**Affiliations:** 1 Heze Medical College, Heze, Shandong, China; 2 Pukyong National University, Busan, Republic of Korea

**Keywords:** academic motivation, Chile, deep learning techniques, differential item functioning, Ecuador, higher education, measurement invariance, self-regulated learning

## Abstract

Meaningful cross-national comparisons of self-regulated learning (SRL), motivation, and related learning-regulation constructs require evidence that the measurement model operates comparably across groups. We tested this requirement for three GAEU-1 dimensions previously retained in Chile and Ecuador using the open Version-5 dataset (*N* = 1,316; Chile *n* = 631; Ecuador *n* = 685). Fifteen ordered indicators representing the GAEU-1 SRL factor, conscious motivational strategies, and deep learning techniques were analyzed with WLSMV, followed by a Wu-Estabrook multigroup ordinal invariance sequence and complementary DIF analyses. Country-specific fit was mixed: CFI and SRMR were relatively favorable, whereas RMSEA indicated residual misspecification, especially in Chile (CFI = 0.947, SRMR = 0.059, RMSEA = 0.094; Ecuador: CFI = 0.952, SRMR = 0.059, RMSEA = 0.085). In the multigroup models, equality constraints on thresholds, loadings, and intercept-related parameters produced no meaningful deterioration in approximate fit, and met the study's prespecified incremental-fit criteria for proceeding to latent comparison within the retained scoring representation. Several items showed FDR-adjusted DIF signals, but none reached the prespecified 0.020 practical-effect criterion. *Post hoc* diagnostics localized the largest residual association to GA7-GA8 in both samples; a one-factor model fit appreciably worse than the retained three-factor representation (Delta CFI = −0.059 in Chile and −0.069 in Ecuador). Polychoric HTMT values were all below 0.85, although Fornell-Larcker diagnostics remained unfavorable for factor pairs involving the GAEU-1 SRL factor. Observed and latent standardized sample contrasts differed only modestly and are interpreted descriptively because the samples differed markedly in university sector and gender composition. The two planned sensitivity analyses and a *post hoc* private-university-only sensitivity analysis preserved the main comparability result. Overall, the three retained GAEU-1 dimensions showed stable measurement parameters across these Chilean and Ecuadorian university samples, while broader structural and country-level interpretations remain outside the evidence provided here.

## 1 Introduction

Self-regulated learning (SRL) describes the processes through which learners plan, monitor, regulate, and adapt cognition, motivation, behavior, and learning conditions in pursuit of academic goals (Zimmerman, 2002; Pintrich, 2004; Panadero, 2017). In higher education, these processes are particularly consequential because students are expected to organize learning resources, sustain effort, respond to difficulty, and engage in strategies that support deeper processing with increasing autonomy.

SRL's conceptual breadth has produced equally broad measurement practice. Available instruments variously emphasize metacognition, motivational regulation, behavioral strategy use, environmental structuring, or combinations of these domains. Consequently, similar labels can refer to different operational definitions. The problem becomes especially important in cross-context research, where score differences may be read as psychological differences even when the measurement model has not been shown to function comparably.

Measurement invariance addresses this problem by testing whether observed indicators relate to latent constructs in sufficiently similar ways across groups. Without such evidence, mean differences may partly reflect group-specific item functioning rather than differences in the target construct (Putnick and Bornstein, 2016). When departures are localized, partial invariance can still permit latent comparisons if adequate anchors remain and any released constraints are justified substantively rather than chosen only to improve fit (Byrne et al., 1989).

For Likert-type items, the issue is not merely technical. Ordered responses arise from thresholds on underlying response propensities, so procedures developed for continuous indicators cannot be transferred without appropriate categorical identification. Ordinal invariance methods explicitly model thresholds and loadings under such constraints (Millsap and Yun-Tein, 2004; Wu and Estabrook, 2016), with practical implementations available in lavaan and semTools (Svetina et al., 2020).

The Learning Management of University Students Scale (GAEU-1) was developed for university students in Chile and Ecuador. Its original 19 items cover self-management/self-regulated learning, conscious motivational strategies, perceived academic performance, and deep learning techniques. In the 2023 validation sample (*N* = 1,373; Chile *n* = 663; Ecuador *n* = 710), perceived academic performance showed the weakest internal consistency, while a reduced representation retaining the other three first-order dimensions fit better in each country and in the pooled sample. The article also reported a second-order general learning-management representation with the same fit. Our analysis takes the three retained dimensions as an inherited scoring framework and asks a narrower question: are their item parameters comparable across the Version-5 Chilean and Ecuadorian samples? It is not a new dimensionality-selection study (Ramos-Galarza et al., 2023).

The original validation study estimated separate country models and reported direct score comparisons, but it did not test multigroup ordinal measurement invariance. A later open Version-5 dataset from the same research program contains 1,316 students (Chile *n* = 631; Ecuador *n* = 685) under a CC BY 4.0 license (Ramos-Galarza et al., 2025b). The public documentation does not establish whether participants overlap with the 2023 sample, so the present work is treated as a distinct secondary psychometric analysis, not an independent replication.

Recent work further shows that SRL measurement structures are instrument- and context-specific. López-Angulo et al. (2024), for example, validated a different three-dimensional SRL instrument in Chilean undergraduates. Regatto-Bonifaz et al. (2025) found cross-national invariance for another self-regulation inventory in Ecuador, Peru, and Venezuela, whereas Wu et al. (2026) reported metric but not scalar invariance for Chinese international and Canadian domestic undergraduates. These differences make direct testing of the GAEU-1 necessary before its scores are compared across the Chilean and Ecuadorian samples.

We examined whether the three retained GAEU-1 dimensions share a sufficiently common measurement basis across the two university samples. The analysis proceeded from country-specific ordinal CFA to a staged Wu-Estabrook multigroup invariance sequence, with equality-constraint diagnostics and DIF analyses used to localize possible item-level differences. The retained three-dimensional representation was inherited from prior validation rather than selected anew here. Accordingly, the central claim concerns cross-sample parameter comparability conditional on that scoring representation, not dimensional optimality. Observed-vs.-latent sample contrasts were examined only after adequate invariance was established and served as a secondary illustration of measurement adjustment.

H1 proposed, on theory-informed grounds, that the correlated representation of the three retained dimensions would be sufficiently defensible in both samples to justify conditional invariance testing. This hypothesis and the analysis protocol were finalized after preliminary feasibility diagnostics and were not preregistered. The achieved invariance level, item-specific DIF, any partial constraints, and the direction of later sample contrasts were therefore treated as planned exploratory outcomes. RQ1 addressed configural, threshold, loading, and intercept-related invariance; RQ2 addressed statistically detectable and non-negligible DIF; and RQ3 specified a partial-invariance fallback if the planned strong/scalar stage was not retained. Structural and discriminant-validity diagnostics added after the primary analysis plan had been finalized are explicitly identified as *post hoc*.

## 2 Materials and methods

### 2.1 Study design and data source

This secondary analysis used the publicly available Learning Strategies in Higher Education: A Research Dataset, Version 5 (doi: 10.17632/7vgyndhb87.5; Ramos-Galarza et al., 2025b), distributed under a Creative Commons Attribution 4.0 International license. No participants were newly recruited and no new data were collected. Version 5 contains *N* = 1,316 (Chile *n* = 631; Ecuador *n* = 685), compared with *N* = 1,373 in the 2023 validation article (Chile *n* = 663; Ecuador *n* = 710). Because the public records do not establish participant overlap, we do not describe the present sample as an independent replication.

The source documentation reports written informed consent and approval by the Ethics Committee for Research Involving Human Beings at the Pontifical Catholic University of Ecuador (approval 2019-58EO; 24 January 2019; Ramos-Galarza et al., 2025a). Preliminary feasibility diagnostics had been reviewed before the analysis plan was locked. The retained three-dimensional representation was evaluated under a theory-informed hypothesis, while the eventual invariance level, item-specific DIF, partial constraints, and latent-mean directions remained exploratory.

### 2.2 Participants

The dataset includes 1,316 undergraduates: 631 from Chile (47.9%) and 685 from Ecuador (52.1%). Mean age was 20.45 years (*SD* = 2.04); 746 participants were women (56.7%) and 570 were men (43.3%) (Ramos-Galarza et al., 2025a). No national probability sampling frame is documented, so neither sample is treated as nationally representative. University-sector composition also differed markedly (private university: Chile 57.5%, Ecuador 98.4%). Institution identifiers are not available in Version 5, precluding institution-clustered or institution-matched analyses. Country functions here as a grouping variable in the measurement models, not as a direct measure of culture. Numeric country codes were mapped from the SPSS value labels.

### 2.3 Measure: GAEU-1

The GAEU-1 contains 19 self-report items rated from 1 (strongly disagree) to 5 (strongly agree). The analysis model retained 15 indicators from three dimensions supported in the earlier validation: the GAEU-1 SRL factor (GA2, GA3, GA4, GA6, GA13, GA14), conscious learning motivation strategies (GA7-GA11), and deep learning techniques (GA16-GA19). Four perceived-academic-performance items (GA1, GA5, GA12, GA15) were used only descriptively because that dimension had shown weaker reliability and the reduced three-dimensional representation fit better in the original study (Ramos-Galarza et al., 2023). The instrument was administered in Spanish in both countries; English wording in the public files is a reporting translation. The GAEU-1 SRL factor captures only part of the broader SRL domain, and the analysis does not resolve whether this three-dimensional representation is the optimal structure. Item wording and assignments appear in Supplementary Table S1.

### 2.4 Data quality assessment

Before structural modeling, responses were checked for missing values, out-of-range scores, category distributions, zero within-person variance across all 19 items, and repeated 19-item response patterns. Uniform responding or a shared Likert pattern was not automatically treated as careless or duplicated because either can occur in valid records. All eligible observations entered the primary analysis. A planned sensitivity analysis repeated the central models after removing respondents with zero within-person variance.

### 2.5 Descriptive statistics and reliability

Descriptive statistics were calculated by country and for the pooled sample. Internal consistency was summarized with coefficient alpha, for comparability with earlier GAEU-1 work, and model-based omega from the standardized ordinal CFA solution. Reliability estimates complemented the invariance tests.

### 2.6 Ordinal CFA of the retained measurement representation

All statistical models were estimated in R using lavaan and semTools; the 15 retained indicators were treated as ordered categorical variables. Models used WLSMV with theta parameterization. The correlated three-dimensional representation was estimated separately in Chile and Ecuador before multigroup testing. This step assessed whether the inherited scoring framework was sufficiently defensible for conditional invariance analysis; it was not intended to select dimensionality anew (Millsap and Yun-Tein, 2004; Wu and Estabrook, 2016; Svetina et al., 2020). The second-order representation reported in the original validation was not treated as an independent fit competitor because a single higher-order factor defined by only three first-order factors is just identified at the higher-order level and therefore adds no independent global-fit test of dimensionality.

Model fit was summarized with the scaled WLSMV chi-square statistic, CFI, TLI, and RMSEA with a 90% confidence interval, and SRMR. Given chi-square sensitivity to sample size, interpretation relied on the pattern across approximate fit indices instead of a single cutoff. Freely estimated factor correlations were reported to show the extent of overlap among dimensions. As *post hoc* discriminant-validity diagnostics added after the primary analysis plan had been finalized, each standardized latent correlation was contrasted with 1 using a Wald statistic based on its model-estimated standard error, and average variance extracted (AVE) was calculated from the completely standardized loadings for the Fornell-Larcker diagnostic (Fornell and Larcker, 1981). These diagnostics assess empirical separation; they do not establish that the retained three-factor representation is uniquely optimal.

### 2.7 Cross-sample ordinal measurement invariance

Cross-sample invariance was tested with multigroup ordinal CFA under the Wu-Estabrook identification framework (Wu and Estabrook, 2016). The pipeline used semTools::measEq.syntax() with standardized latent-factor identification and the Wu-Estabrook strategy for categorical indicators.

The sequence comprised country-specific ordinal CFA, a configural model, equality of item thresholds, equality of thresholds plus factor loadings, and equality of thresholds, loadings, and the intercept-related parameters required by the identified ordinal model. If any stage failed the decision rule, the plan called for the minimum justified partial invariance before proceeding.

Invariance decisions focused on deterioration in approximate fit. The decision guides were a CFI decrease no larger than 0.010 (Delta CFI ≥ −0.010) and an RMSEA increase no larger than 0.015 (Delta RMSEA < = +0.015), interpreted alongside SRMR, scaled nested comparisons, parameter estimates, and local diagnostics (Cheung and Rensvold, 2002; Chen, 2007). An improvement in CFI was not counted against invariance. The same scaled WLSMV fit-index family was used throughout, and the numerical guides were treated as heuristics rather than universal pass/fail cutoffs. Because exact nested chi-square tests can reject small discrepancies in large samples, robust scaled chi-square difference tests are reported alongside, but do not replace, the incremental-fit criteria. Analytical RMSEA power was additionally calculated following MacCallum et al. (1996), contrasting close fit (RMSEA = 0.05) with appreciable misfit (RMSEA = 0.08) at alpha = 0.05 for the country-specific and multigroup models.

### 2.8 Differential item functioning

DIF analysis served as a complementary item-level diagnostic; it was not an independent gatekeeper for partial invariance. Factor-wise iterative hybrid ordinal logistic regression/IRT procedures were implemented with lordif (Choi et al., 2011), using Chile as the reference group and Ecuador as the focal group. Uniform, non-uniform, and total DIF were examined. Because the deep-learning factor has only four indicators, its purification and anchor information were interpreted cautiously and considered together with multigroup diagnostics and item content.

To distinguish statistical detection from practical importance, an item was treated as a non-negligible DIF candidate only when both the Benjamini-Hochberg FDR-adjusted q value was below 0.05 and the change in McFadden pseudo-R-squared reached 0.02 (Benjamini and Hochberg, 1995). The 0.02 effect-size rule was study-specific, not a universal benchmark; significant results below it were described as small under this operational definition. Because retrospective power for an observed DIF effect is not itself evidence of absence, we added a threshold-sensitivity check that reapplied the classification at Delta R-squared thresholds of 0.010, 0.015, and 0.020. This makes explicit how the practical-DIF conclusion changes under more permissive effect-size rules.

### 2.9 Partial invariance

Partial invariance was a contingency for any strong/scalar-stage equality step that failed the decision guides. Constraints were not to be freed automatically from the largest modification index. A release required convergence from at least two of three sources: local equality diagnostics, complementary DIF evidence, and an interpretable item-content or contextual rationale. No single fit statistic was sufficient (Byrne et al., 1989). Residual variances were not constrained across groups, so strict invariance was not tested.

If needed, releases would proceed sequentially from thresholds to loadings to intercept-related parameters, using the smallest defensible set of freed constraints. Latent means would be interpreted only when at least two untouched invariant indicators remained per factor, with every release recorded in a supplementary decision log.

### 2.10 Secondary post-invariance sample contrasts

Latent sample contrasts were estimated only after adequate strong/scalar-level or partial strong/scalar-level invariance had been established. Their purpose was descriptive: to show how measurement modeling changed sample comparisons, not to isolate national, cultural, or institutional effects. Chile was the reference group. Output included the Ecuador-minus-Chile latent mean difference, standard error, 95% confidence interval, and Holm-adjusted *p* value for each retained dimension. Observed Cohen d used the pooled observed SD; latent Cohen d used the pooled model-estimated latent SD. Hedges g was retained as a supplementary observed-score effect size.

The observed-to-latent shift was latent Cohen d minus observed Cohen d. For Figure 1, the confidence interval around latent d was obtained by dividing the model-estimated latent-mean interval by the pooled latent SD, treated as fixed for visualization. Formal inference remained based on the unstandardized latent mean difference and its model-based uncertainty.

**Figure 1:**
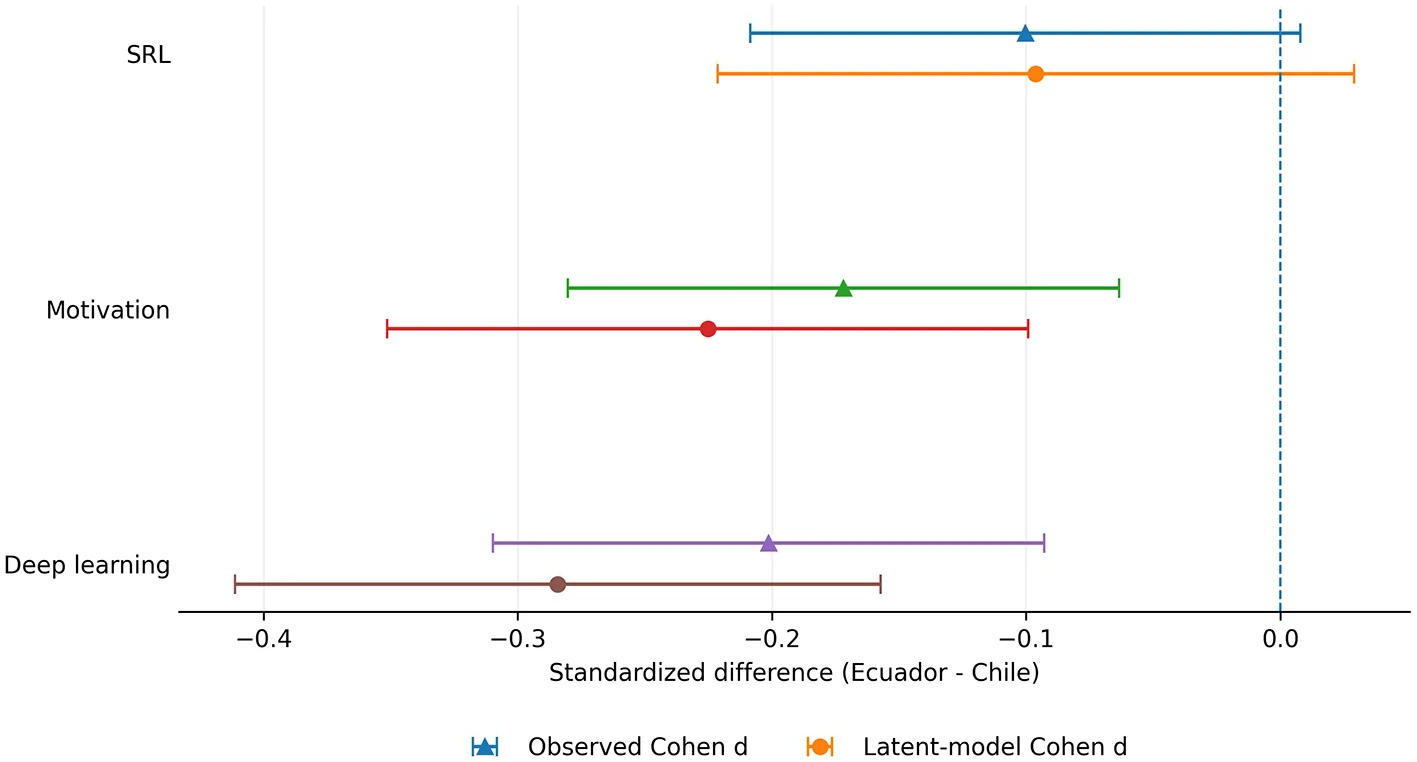
Observed and latent standardized sample contrasts after strong/scalar-level ordinal invariance. Effects are coded Ecuador—Chile. Triangles show observed Cohen d; circles show latent-model Cohen d with approximate 95% confidence intervals. Formal inference uses the model-estimated latent mean difference and Holm-adjusted *p* values. The figure is descriptive and is not interpreted as evidence of national or cultural effects.

### 2.11 Sensitivity analyses

Two sensitivity analyses were included in the analysis plan. One repeated the final measurement model after removing participants with zero within-person variance across all 19 items. The other allowed residual covariances for GA2-GA3 and GA7-GA8 after preliminary, data-informed diagnostics suggested localized dependence. The same invariance specification and latent-effect extraction were then re-estimated. The two residual associations were not theory-prespecified. Their content is nevertheless interpretable: GA2 and GA3 both concern preparation of the study setting, while GA7 and GA8 both involve motivational self-talk in the face of academic demands. Because the links were selected after inspecting preliminary diagnostics, the local-dependence model remains a sensitivity check rather than a revised primary model.

### 2.12 *Post hoc* diagnostic and robustness analyses

Four additional diagnostics were specified as *post hoc* analyses and were kept separate from the locked primary invariance sequence. First, for each country-specific retained three-factor CFA, residual-correlation diagnostics and residual-covariance modification indices were exported to localize the largest remaining areas of misfit; these diagnostics were not used to revise the primary model or to retrospectively redefine the invariance decision rules. Second, an intentionally parsimonious 15-item one-factor ordinal CFA was fit separately in Chile and Ecuador and compared descriptively with the retained correlated three-factor representation using scaled chi-square, degrees of freedom, CFI, TLI, RMSEA, and SRMR. Because the one-factor and three-factor models are competing dimensional representations rather than an invariance sequence, their comparison is interpreted through the pattern of fit indices rather than as an adjacent equality-constraint test. Third, polychoric HTMT values were calculated for each factor pair as an additional discriminant-validity diagnostic and interpreted alongside the Wald and Fornell-Larcker results (Henseler et al., 2015); the conventional 0.85 benchmark was used only as a descriptive heuristic. Fourth, the source university-type labels yielded a private-university subset of *N* = 1,037 (Chile *n* = 363; Ecuador *n* = 674), and the full M1-M4 ordinal invariance sequence was repeated in that restricted sample. This private-only analysis was treated solely as a composition-sensitivity check: it cannot create institutionally matched samples, and it changes the composition of the Chilean group much more than the Ecuadorian group. All four analyses were conducted after the primary analysis plan had been finalized, were not preregistered, and were not allowed to alter the prespecified primary model, DIF threshold, or the interpretation of the primary sample contrasts.

### 2.13 Reproducibility

All analyses were run through scripted R workflows. The reproducibility archive contains the code, package versions, model syntax, session information, invariance and DIF output, final parameter estimates, sensitivity results, and analysis-ready tables and figures. The workflow did not automate modification-index chasing for partial invariance.

## 3 Results

*Analyses followed the scripted R workflow defined in Analysis Protocol V1.2*.

### 3.1 Sample characteristics and data quality

The analytic sample comprised 1,316 students (Chile *n* = 631; Ecuador *n* = 685). All 19 GAEU-1 items were complete and within the permitted 1–5 range. Nine participants (0.68%) had zero within-person variance across all 19 items. Fifty-four shared a complete 19-item response pattern with at least one other participant; these records were retained because an identical Likert pattern alone does not establish duplication or invalid responding. Sample composition differed appreciably: women represented 46.9% of the Chilean sample and 65.7% of the Ecuadorian sample, while private-university students represented 57.5% and 98.4%, respectively (Table 1). Removing the nine zero-variance cases (n = 1,307) did not materially change the results.

**Table 1:** Sample characteristics by country.

Characteristic	Chile (*n* = 631)	Ecuador (*n* = 685)	Total (*N* = 1,316)
Age, *M* (SD)	20.18 (2.06)	20.71 (1.99)	20.45 (2.04)
Female, *n* (%)	296 (46.9%)	450 (65.7%)	746 (56.7%)
Male, *n* (%)	335 (53.1%)	235 (34.3%)	570 (43.3%)
Private university, *n* (%)	363 (57.5%)	674 (98.4%)	1037 (78.8%)
State university, *n* (%)	268 (42.5%)	9 (1.3%)	277 (21.0%)
Living with family members, *n* (%)	473 (75.0%)	613 (89.5%)	1086 (82.5%)

University type was unspecified for two Ecuador respondents; percentages are based on the full country denominators.

### 3.2 Descriptive statistics and reliability

Internal consistency was adequate across the three analyzed dimensions. In the pooled sample, alpha was 0.782 for the GAEU-1 SRL factor, 0.793 for conscious motivational strategies, and 0.753 for deep learning; corresponding omega estimates were 0.837, 0.890, and 0.806. Country-specific omega values ranged from 0.764 to 0.905. The supplementary perceived-academic-performance composite was weaker (alpha = 0.610), consistent with its exclusion from the invariance model (Table 2).

**Table 2:** Descriptive statistics and reliability of GAEU-1 constructs.

Construct	Items	M	SD	Alpha	Omega	Chile omega	Ecuador omega
Self-regulated learning	6	4.051	0.635	0.782	0.837	0.847	0.831
Conscious learning motivation strategies	5	4.111	0.715	0.793	0.890	0.905	0.875
Deep learning techniques	4	3.816	0.779	0.753	0.806	0.845	0.764
Perceived academic performance^*^	4	3.869	0.644	0.610	-	-	-

^*^Supplementary construct; not included in the primary three-factor invariance model.

### 3.3 Country-specific ordinal CFA

The correlated three-dimensional representation was estimated separately by country with WLSMV. Fit was mixed. In Chile, chi-square(87) = 575.36, CFI = 0.947, TLI = 0.937, RMSEA = 0.094, 90% CI [.087, 0.102], and SRMR = 0.059; in Ecuador, chi-square(87) = 512.95, CFI = 0.952, TLI = 0.942, RMSEA = 0.085, 90% CI [.078, 0.092], and SRMR = 0.059. Standardized loadings were positive throughout (0.506–0.898 in Chile; 0.491–0.898 in Ecuador). Analytical close-fit power (RMSEA = 0.05 vs. 0.08, alpha = 0.05) exceeded 0.999 in both country models, indicating that the elevated RMSEA values cannot reasonably be attributed to low statistical sensitivity. The data-informed local-dependence sensitivity model improved global fit (CFI = 0.966, TLI = 0.958, RMSEA = 0.074, SRMR = 0.052) without changing the invariance result or the later sample-contrast pattern. H1 therefore received partial support: the inherited representation was usable for conditional invariance testing, but baseline absolute fit showed non-trivial misspecification.

### 3.4 Ordinal measurement invariance

The configural model yielded CFI = 0.950, TLI = 0.939, RMSEA = 0.089, and SRMR = 0.059. Constraining thresholds changed CFI by −0.001 while RMSEA improved by 0.006. Adding loading equality improved both CFI (+0.005) and RMSEA (−0.006), and adding the intercept-related constraints changed CFI by less than 0.001 while RMSEA improved by a further 0.002. All planned stages through the strong/scalar level met the incremental decision rule, and no partial constraints were needed (Table 3). Robust scaled difference tests were statistically significant at each restriction step (threshold vs. configural: Delta chi-square = 54.47, Delta *df* = 30, *p* = 0.004; loading vs. threshold: Delta chi-square = 27.06, Delta *df* = 12, *p* = 0.008; intercept-related vs. loading: Delta chi-square = 54.76, Delta *df* = 12, *p* < 0.001), illustrating the greater sensitivity of exact-fit tests relative to the small changes in approximate fit. Lavaan reported 156, 186, 189, and 177 model parameters for M1-M4, respectively (Supplementary Table S10). Analytical RMSEA power exceeded 0.999 for all four multigroup stages. Residual variances remained freely estimated across groups; strict invariance was not tested. Factor correlations in the final model were substantial (Supplementary Table S9).

**Table 3:** Cross-sample ordinal measurement invariance (decision guides are heuristic, not universal cutoffs; robust scaled difference tests and model-parameter counts are reported in Supplementary Table S10).

Model	Constraints	CFI	TLI	RMSEA	SRMR	Delta CFI	Delta RMSEA	Incremental-fit result	Chi-square (*df*)
M1	Configural	0.950	0.939	0.089	0.059	-	-	Meets guide	1084.88 (174)
M2	Thresholds	0.948	0.947	0.083	0.059	−0.001	−0.006	Meets guide	1136.11 (204)
M3	Thresholds + loadings	0.954	0.955	0.077	0.060	+0.005	−0.006	Meets guide	1055.24 (216)
M4	Thresholds + loadings + intercepts	0.953	0.957	0.075	0.060	−0.000	−0.002	Meets guide	1072.27 (228)
M5	Partial invariance	-	-	-	-	-	-	Not required	-

### 3.5 Differential item functioning

Several indicators showed statistically detectable DIF after FDR correction, but none met the joint criterion for non-negligible DIF (*q* < 0.05 and Delta McFadden R-squared ≥ 0.02). Total-DIF signals appeared for eight items (GA2, GA3, GA4, GA6, GA7, GA13, GA18, GA19), seven showed uniform-DIF signals, and GA7 alone showed a non-uniform signal. The largest total Delta R-squared was 0.0103 for GA3; this total is the sum, to rounding, of 0.0092 uniform and 0.0011 non-uniform components. The largest non-uniform value was 0.0040 for GA7. Threshold sensitivity showed that GA3 would cross a 0.010 practical-effect rule, whereas no item crossed 0.015 or 0.020. Thus, the statement that no item showed non-negligible DIF is specific to the prespecified 0.020 rule and should not be read as proof of zero DIF. All 15 indicators were retained under the planned criterion (Table 4; Supplementary Tables S4, S12).

**Table 4:** Statistically detectable DIF signals under the strong/scalar-level model; all effects remained below the study's practical-effect threshold.

Item	Factor	Detectable DIF signal	Smallest BH-FDR q	Max delta McFadden R2	Non-negligible DIF?	MGCFA evidence	Final treatment
GA18	DEEP	Uniform/total	0.005	0.0036	No	Supported	Retained
GA19	DEEP	Uniform/total	0.007	0.0027	No	Supported	Retained
GA7	MOT	Total + non-uniform	0.002	0.0042	No	Supported	Retained
GA13	SRL	Uniform/total	0.011	0.0034	No	Supported	Retained
GA2	SRL	Uniform/total	0.007	0.0029	No	Supported	Retained
GA3	SRL	Uniform/total	< 0.001	0.0103	No	Supported	Retained
GA4	SRL	Uniform/total	0.018	0.0026	No	Supported	Retained
GA6	SRL	Uniform/total	0.026	0.0028	No	Supported	Retained

### 3.6 Partial invariance

Partial invariance was not required under the planned incremental-fit rule. Thresholds, loadings, and intercept-related parameters remained constrained across groups for all 15 indicators. Residual variances were free, so the result corresponds only to the strong/scalar-level ordinal stage tested here; strict invariance was not tested. This is a statement about equality constraints within the retained representation, not a claim that the underlying measurement model is optimal.

### 3.7 Secondary post-invariance sample contrasts

Table 5 and Figure 1 compare observed and latent standardized sample contrasts after invariance was established. With effects coded Ecuador minus Chile, observed Cohen d values were −0.100 for the GAEU-1 SRL factor, −0.172 for motivation, and −0.201 for deep learning. The latent SRL contrast remained small and non-significant (latent *d* = −0.096; latent mean difference = −0.095, 95% CI [−0.219, 0.029], Holm-adjusted *p* = 0.132). Latent *d* was −0.225 for conscious motivation and −0.284 for deep learning (both Holm-adjusted *p* < 0.001). Relative to the observed composites, the standardized shifts were +0.004, −0.053, and −0.083 SD. Results were similar after removing zero-variance cases (latent *d* = −0.099, −0.228, −0.292) and in the local-dependence sensitivity model (−0.092, −0.233, −0.284). Because the two samples differed markedly in gender and university sector, these values are treated as descriptive sample contrasts rather than estimates of country, cultural, or institutional effects.

**Table 5:** Observed and latent standardized sample contrasts after strong/scalar-level invariance.

Construct	Observed Cohen d	Latent mean difference	95% CI	Latent Cohen *d*	Holm-adjusted *p*	Latent *d*—observed *d*
Self-regulated learning	−0.100	−0.095	[−0.219, 0.029]	−0.096	0.132	+0.004
Conscious motivation	−0.172	−0.215	[−0.335, −0.095]	−0.225	< 0.001	−0.053
Deep learning	−0.201	−0.256	[−0.371, −0.142]	−0.284	< 0.001	−0.083

Effects are coded Ecuador—Chile; positive values favor Ecuador and negative values favor Chile. The last column is latent Cohen d minus observed Cohen *d*. These are descriptive sample contrasts after measurement adjustment, not estimates of isolated country effects.

### 3.8 *Post hoc* diagnostic and robustness analyses

The *post hoc* country-specific diagnostics localized the strongest residual dependence to GA7-GA8 in both samples. In Chile, the residual correlation was 0.174 (standardized residual *z* = 8.46) and the corresponding residual-covariance modification index was 91.85 (EPC = 0.596); in Ecuador, the residual correlation was 0.201 (*z* = 8.64) and the modification index was 79.38 (EPC = 0.461). GA2-GA3 was also among the strongest local associations, with the second-largest residual-covariance modification index in both countries (35.78 in Chile; 24.78 in Ecuador). These patterns converge with the two data-informed residual links examined in the local-dependence sensitivity model, but they were not used to alter the primary specification.

The *post hoc* one-factor model fit appreciably worse than the retained correlated three-factor representation in both countries. In Chile, the one-factor model yielded CFI = 0.888, TLI = 0.869, RMSEA = 0.135, and SRMR = 0.088, compared with 0.947, 0.937, 0.094, and 0.059 for the retained model (Delta CFI = −0.059; Delta RMSEA = +0.041). In Ecuador, the corresponding one-factor values were CFI = 0.883, TLI = 0.863, RMSEA = 0.130, and SRMR = 0.089, compared with 0.952, 0.942, 0.085, and 0.059 (Delta CFI = −0.069; Delta RMSEA = +0.045). This comparison argues against simple unidimensionality in these samples but does not establish that the retained three-factor representation is uniquely optimal.

Polychoric HTMT values ranged from 0.695 to 0.836 in Chile and from 0.609 to 0.767 in Ecuador; all six values were below 0.85. This diagnostic is more favorable than the Fornell-Larcker results, which were not met for factor pairs involving the GAEU-1 SRL factor. Taken together, the diagnostics indicate that the factors are not empirically identical, while the strength of discriminant separation remains criterion-dependent rather than uniformly compelling.

The private-university-only sensitivity analysis reproduced the primary invariance pattern. In the restricted sample (*N* = 1,037; Chile *n* = 363; Ecuador *n* = 674), M1 yielded CFI = 0.949, TLI = 0.939, RMSEA = 0.089, and SRMR = 0.062. Relative to the preceding stage, M2 changed CFI by −0.001 and RMSEA by −0.006; M3 changed CFI by +0.005 and RMSEA by −0.006; and M4 changed CFI by less than +0.001 and RMSEA by −0.002. The final M4 fit was CFI = 0.954, TLI = 0.957, RMSEA = 0.074, and SRMR = 0.062. Standardized latent mean estimates for Ecuador relative to Chile were −0.071 for the GAEU-1 SRL factor, −0.278 for motivation, and −0.273 for deep learning. The restriction therefore preserved the measurement-comparability conclusion and the broad contrast pattern, but it did not create institutionally or compositionally matched groups.

## 4 Discussion

### 4.1 Principal findings

This study asked a focused measurement question: whether three GAEU-1 dimensions retained in earlier validation work functioned comparably across the Version-5 Chilean and Ecuadorian samples. The answer is conditional rather than absolute. Country-specific fit showed non-trivial misspecification, particularly in RMSEA, and the dimensions overlapped substantially. At the same time, imposing threshold, loading, and intercept-related equality produced little deterioration in approximate multigroup fit, DIF effects were small under the prespecified practical criterion, and the two planned sensitivity analyses preserved the same comparability conclusion. The evidence therefore supports parameter stability within the inherited scoring representation, not confirmation that the representation itself is the best structural model. Post-invariance sample contrasts remain a secondary illustration rather than evidence of country-level differences.

### 4.2 What the invariance result establishes

Within-sample model adequacy and cross-sample parameter equivalence answer different questions, but the former sets an important boundary on the latter. Here, RMSEA remained above conventional close-fit values in both country models, and analytical power was more than sufficient to detect appreciable misspecification. We therefore do not use invariance to “rescue” poor absolute fit. Instead, the result is interpreted conditionally: given the inherited three-dimensional scoring representation, adding equality constraints did not materially worsen CFI or RMSEA. Robust scaled chi-square difference tests were nevertheless significant at every restriction step, reinforcing that exact equality is an unnecessarily strong description in this sample size. The strong/scalar-level result should therefore be read as approximate parameter stability at the level tested, not evidence of strict invariance, dimensional optimality, or nationally representative equivalence.

### 4.3 Item-level DIF was statistically detectable but small

The DIF analysis adds an item-level view of the same pattern. Several tests remained significant after FDR adjustment, yet the largest total McFadden Delta *R*-squared was 0.0103. Under the prespecified 0.020 practical-effect rule, no item required release from the multigroup constraints. A *post hoc* threshold sensitivity analysis makes the dependence on that rule explicit: GA3 would be flagged at 0.010, whereas no item reaches 0.015 or 0.020. Thus, the data support small rather than absent item-level differences. This distinction is especially important because a null or sub-threshold DIF result is not equivalent to a formal demonstration of no effect. Equivalence-testing approaches with prospectively justified bounds may be useful in future educational measurement work when the research goal is to establish practical equivalence rather than merely fail to detect a difference (Ponce-Renova, 2022).

### 4.4 Factor overlap and local dependence

The three dimensions were strongly related, most notably the GAEU-1 SRL and deep-learning factors in Chile (*r* = 0.836). *Post hoc* discriminant diagnostics sharpen this limitation. Wald contrasts showed that all six country-specific factor correlations were statistically below 1 (all |z| ≥ 8.88, *p* < 0.001), so the factors were not empirically identical. Polychoric HTMT values were also below the conventional 0.85 heuristic in both countries (Chile 0.695–0.836; Ecuador 0.609–0.767), which provides comparatively favorable evidence of distinction (Henseler et al., 2015). However, the Fornell-Larcker diagnostic was less reassuring: the square root of AVE for SRL was 0.696 in Chile and 0.671 in Ecuador, below its correlations with both motivation and deep learning; only the motivation-deep-learning pair satisfied the criterion in both samples. The *post hoc* one-factor CFA fit appreciably worse than the retained correlated three-factor model in each country (Delta CFI = −0.059 and −0.069; Delta RMSEA = +0.041 and +0.045 for Chile and Ecuador, respectively), arguing against simple unidimensionality. The criteria nevertheless do not establish unique dimensional optimality. A more complex hierarchical, bifactor, or other competing representation remains plausible, and stronger dimensionality evidence would require prospectively specified alternatives evaluated in independent or cross-validated data.

### 4.5 Interpreting the secondary sample contrasts

The observed and latent standardized contrasts were close for the GAEU-1 SRL factor and somewhat farther apart for motivation and deep learning. Latent modeling changed the magnitude of the latter contrasts modestly, but it did not transform their overall scale. More importantly, the analysis cannot explain why the samples differ. University-sector composition was highly unequal (98.4% private in Ecuador vs. 57.5% in Chile), and gender distributions also differed. The private-university-only sensitivity analysis reproduced the strong/scalar-level incremental-fit result (final M4: CFI = 0.954, TLI = 0.957, RMSEA = 0.074, SRMR = 0.062) and yielded a broadly similar latent-contrast pattern (standardized estimates: SRL = −0.071, motivation = −0.278, deep learning = −0.273). That robustness check does not remove compositional confounding: it excludes 42.5% of the Chilean sample but only 1.6% of the Ecuadorian sample, retains a marked gender imbalance, and cannot create institutionally matched groups because institution identifiers are unavailable. The latent contrasts therefore remain descriptive of these two samples after measurement adjustment, not estimates of national, cultural, or sector effects.

### 4.6 Implications for SRL measurement

For Latin American higher-education research, the main implication is construct-specific rather than a generic claim about SRL. The GAEU-1 SRL factor emphasizes study preparation, environmental structuring, autonomous responsibility, time management, and problem solving; it does not cover the full planning-monitoring-reflection cycle represented in broader SRL theories. Both national samples completed the instrument in Spanish, so the present cross-sample analysis is not confounded by different administration languages. Within that shared-language context, the results indicate that the retained GAEU-1 thresholds and loadings can be treated as approximately stable across these particular Chilean and Ecuadorian university samples, conditional on the inherited representation. This is useful for researchers using GAEU-1 in similar Latin American university settings, but it does not establish regional invariance across Latin America. Future studies should recruit institutionally diverse samples, retain institution identifiers, preregister structural alternatives, and test whether the same parameter stability extends to additional countries and university sectors.

### 4.7 Limitations

The sampling design places the clearest limits on interpretation. Neither country sample was drawn from a documented national probability frame, and the groups differed sharply in university sector and gender. Only nine Ecuadorian respondents were from state universities; two Ecuadorian records had missing university-type labels. The private-university-only sensitivity analysis included 1,037 respondents (Chile *n* = 363; Ecuador *n* = 674) and reproduced the main incremental invariance conclusion, but it did not produce institutionally matched groups and materially redefined the Chilean sample. Gender imbalance also persisted within that restriction (women: Chile 35.8%; Ecuador 66.0%). Institution identifiers were unavailable, so clustering and institution-level matching could not be modeled. The post-invariance contrasts describe these samples only and cannot disentangle sample composition from broader country context. Two-country findings should not be generalized to Latin American higher education as a whole, and country itself is too coarse to stand in for culture, pedagogy, or socioeconomic context.

Measurement limitations are equally important. Several items were concentrated in the upper response categories, which may reduce discrimination among students at higher levels even when ordinal modeling is used. The Version-5 data do not contain respondent-level evidence on item interpretation, although cognitive interviews were reported during the original scale-development study (Ramos-Galarza et al., 2023). The cross-sectional design supports measurement-comparability conclusions but not developmental or causal claims. The local-dependence specification was data-informed and remains a sensitivity analysis. The three-dimensional scoring representation was inherited from prior work rather than selected in this study. Although the *post hoc* one-factor model fit substantially worse, the Fornell-Larcker results and high latent correlations show incomplete discriminant separation, while HTMT was more favorable; these mixed diagnostics do not establish that the retained representation is uniquely optimal. Finally, the practical-DIF conclusion depends on an operational effect-size threshold; threshold sensitivity shows that GA3 would be flagged under a 0.010 rule. These boundaries favor cautious language about approximate comparability rather than claims of structural or practical equivalence. All one-factor, residual-diagnostic, HTMT, and private-only analyses are *post hoc* analyses of the same public dataset and should be interpreted as diagnostic or robustness checks rather than independent confirmation.

## 5 Conclusion

Measurement comparability should precede substantive interpretation of cross-sample score differences. In these Chilean and Ecuadorian university samples, the three retained GAEU-1 dimensions met the planned strong/scalar-level ordinal incremental-fit criteria, and no item reached the prespecified 0.020 practical-DIF threshold. This supports approximate cross-sample parameter stability conditional on the retained scoring representation. The conclusion is deliberately bounded: country-specific RMSEA indicated non-trivial misspecification, exact nested tests rejected equality constraints, and discriminant evidence was mixed. A *post hoc* one-factor model fit appreciably worse than the retained representation, but that comparison does not establish a uniquely optimal dimensional structure. The private-university-only sensitivity analysis reproduced the invariance conclusion without resolving the samples' broader compositional imbalance. Post-invariance contrasts therefore remain sample-descriptive. The evidence supports cautious model-based use of the retained dimensions in these samples while keeping structural, equivalence, and country-level claims separate from what was actually tested.

## Data Availability

Publicly available datasets were analyzed in this study. This data can be found here: Mendeley Data, Version 5: https://doi.org/10.17632/7vgyndhb87.5.
