# Supplementary material for: Ordinal measurement invariance of three retained GAEU-1 dimensions in Chilean and Ecuadorian university samples
Source: Front Psychol. 2026 Oct 15;17:1990515. doi: 10.3389/fpsyg.2026.1990515 (PMC13716558; doi:10.3389/fpsyg.2026.1990515)
Supplement: Supplementary file 1 [file Supplementary_file_1.docx]

Supplementary Material

# Supplementary Table S1. GAEU-1 item wording, factor assignment, and analytic role

| **Item** | **Item wording** | **Factor** | **Role** |
| --- | --- | --- | --- |
| GA1 | I think that my academic performance is the same or better than that of my partners. | Perceived academic performance | Supplementary |
| GA2 | Before studying, I organize the materials I will need (like notebooks, books, notes, or others). | Self-regulated learning | Primary |
| GA3 | To study, I look for a quiet, well-illuminated and comfortable place. | Self-regulated learning | Primary |
| GA4 | I focus my learning on an autonomous way without depending on someone to help me achieve it. | Self-regulated learning | Primary |
| GA5 | I believe that my academic performance is better than what my grades show. | Perceived academic performance | Supplementary |
| GA6 | I live up to my academic university responsibilities in an efficient way. | Self-regulated learning | Primary |
| GA7 | In order to finish my academic assignments, I tell myself encouraging words to feel motivated. | Conscious learning motivation strategies | Primary |
| GA8 | When I go through academic difficulties, I tell myself I am able to overcome them and move on. | Conscious learning motivation strategies | Primary |
| GA9 | I keep myself optimistic and willing to learn. | Conscious learning motivation strategies | Primary |
| GA10 | I keep myself motivated to learn because I have future plans. | Conscious learning motivation strategies | Primary |
| GA11 | I foresee my academic future positively and with motivation. | Conscious learning motivation strategies | Primary |
| GA12 | I consider that my academic performance is satisfactory. | Perceived academic performance | Supplementary |
| GA13 | I organize my time to fulfil my academic university tasks. | Self-regulated learning | Primary |
| GA14 | I am able to find solutions to the problems that I go through during the process of learning at university. | Self-regulated learning | Primary |
| GA15 | I consider I have the ability to learn all the contents of my career's contents. | Perceived academic performance | Supplementary |
| GA16 | I use summaries, outlines, conceptual maps; I highlight ideas, I do critical reading, and I use other resources to learn significantly. | Deep learning techniques | Primary |
| GA17 | When I finish each task or content, I verify what I learned and what I need to review. | Deep learning techniques | Primary |
| GA18 | When the content or topic is difficult, I check it again. | Deep learning techniques | Primary |
| GA19 | I am used to investigating the contents of the subjects on my own without it being an obligation. | Deep learning techniques | Primary |

Response scale: 1 = Strongly disagree; 2 = Moderately disagree; 3 = Neither agree nor disagree; 4 = Moderately agree; 5 = Strongly agree. Wording reproduced from the open-data questionnaire supplied with the dataset.

# Supplementary Table S2. Ordered response distributions by country

| **Item** | **Country** | **1 n (%)** | **2 n (%)** | **3 n (%)** | **4 n (%)** | **5 n (%)** |
| --- | --- | --- | --- | --- | --- | --- |
| GA1 | Chile | 41 (6.5%) | 66 (10.5%) | 239 (37.9%) | 208 (33.0%) | 77 (12.2%) |
| GA2 | Chile | 14 (2.2%) | 33 (5.2%) | 84 (13.3%) | 230 (36.5%) | 270 (42.8%) |
| GA3 | Chile | 8 (1.3%) | 17 (2.7%) | 73 (11.6%) | 190 (30.1%) | 343 (54.4%) |
| GA4 | Chile | 8 (1.3%) | 28 (4.4%) | 114 (18.1%) | 242 (38.4%) | 239 (37.9%) |
| GA5 | Chile | 16 (2.5%) | 44 (7.0%) | 174 (27.6%) | 244 (38.7%) | 153 (24.2%) |
| GA6 | Chile | 5 (0.8%) | 18 (2.9%) | 89 (14.1%) | 302 (47.9%) | 217 (34.4%) |
| GA7 | Chile | 66 (10.5%) | 63 (10.0%) | 146 (23.1%) | 178 (28.2%) | 178 (28.2%) |
| GA8 | Chile | 19 (3.0%) | 29 (4.6%) | 100 (15.8%) | 237 (37.6%) | 246 (39.0%) |
| GA9 | Chile | 5 (0.8%) | 7 (1.1%) | 64 (10.1%) | 221 (35.0%) | 334 (52.9%) |
| GA10 | Chile | 5 (0.8%) | 9 (1.4%) | 48 (7.6%) | 178 (28.2%) | 391 (62.0%) |
| GA11 | Chile | 4 (0.6%) | 12 (1.9%) | 69 (10.9%) | 179 (28.4%) | 367 (58.2%) |
| GA12 | Chile | 16 (2.5%) | 41 (6.5%) | 162 (25.7%) | 247 (39.1%) | 165 (26.1%) |
| GA13 | Chile | 20 (3.2%) | 45 (7.1%) | 148 (23.5%) | 253 (40.1%) | 165 (26.1%) |
| GA14 | Chile | 1 (0.2%) | 23 (3.6%) | 105 (16.6%) | 307 (48.7%) | 195 (30.9%) |
| GA15 | Chile | 2 (0.3%) | 20 (3.2%) | 93 (14.7%) | 240 (38.0%) | 276 (43.7%) |
| GA16 | Chile | 16 (2.5%) | 48 (7.6%) | 104 (16.5%) | 183 (29.0%) | 280 (44.4%) |
| GA17 | Chile | 22 (3.5%) | 48 (7.6%) | 162 (25.7%) | 227 (36.0%) | 172 (27.3%) |
| GA18 | Chile | 7 (1.1%) | 18 (2.9%) | 78 (12.4%) | 230 (36.5%) | 298 (47.2%) |
| GA19 | Chile | 46 (7.3%) | 59 (9.4%) | 189 (30.0%) | 194 (30.7%) | 143 (22.7%) |
| GA1 | Ecuador | 17 (2.5%) | 45 (6.6%) | 200 (29.2%) | 256 (37.4%) | 167 (24.4%) |
| GA2 | Ecuador | 17 (2.5%) | 59 (8.6%) | 133 (19.4%) | 224 (32.7%) | 252 (36.8%) |
| GA3 | Ecuador | 6 (0.9%) | 39 (5.7%) | 123 (18.0%) | 237 (34.6%) | 280 (40.9%) |
| GA4 | Ecuador | 7 (1.0%) | 31 (4.5%) | 113 (16.5%) | 254 (37.1%) | 280 (40.9%) |
| GA5 | Ecuador | 13 (1.9%) | 29 (4.2%) | 186 (27.2%) | 265 (38.7%) | 192 (28.0%) |
| GA6 | Ecuador | 3 (0.4%) | 27 (3.9%) | 121 (17.7%) | 267 (39.0%) | 267 (39.0%) |
| GA7 | Ecuador | 73 (10.7%) | 101 (14.7%) | 173 (25.3%) | 186 (27.2%) | 152 (22.2%) |
| GA8 | Ecuador | 21 (3.1%) | 35 (5.1%) | 141 (20.6%) | 239 (34.9%) | 249 (36.4%) |
| GA9 | Ecuador | 5 (0.7%) | 22 (3.2%) | 85 (12.4%) | 263 (38.4%) | 310 (45.3%) |
| GA10 | Ecuador | 5 (0.7%) | 16 (2.3%) | 77 (11.2%) | 209 (30.5%) | 378 (55.2%) |
| GA11 | Ecuador | 6 (0.9%) | 23 (3.4%) | 76 (11.1%) | 213 (31.1%) | 367 (53.6%) |
| GA12 | Ecuador | 9 (1.3%) | 26 (3.8%) | 129 (18.8%) | 288 (42.0%) | 233 (34.0%) |
| GA13 | Ecuador | 8 (1.2%) | 60 (8.8%) | 168 (24.5%) | 268 (39.1%) | 181 (26.4%) |
| GA14 | Ecuador | 3 (0.4%) | 15 (2.2%) | 142 (20.7%) | 305 (44.5%) | 220 (32.1%) |
| GA15 | Ecuador | 8 (1.2%) | 25 (3.6%) | 95 (13.9%) | 271 (39.6%) | 286 (41.8%) |
| GA16 | Ecuador | 17 (2.5%) | 49 (7.2%) | 154 (22.5%) | 240 (35.0%) | 225 (32.8%) |
| GA17 | Ecuador | 23 (3.4%) | 80 (11.7%) | 201 (29.3%) | 244 (35.6%) | 137 (20.0%) |
| GA18 | Ecuador | 8 (1.2%) | 32 (4.7%) | 147 (21.5%) | 261 (38.1%) | 237 (34.6%) |
| GA19 | Ecuador | 30 (4.4%) | 80 (11.7%) | 216 (31.5%) | 233 (34.0%) | 126 (18.4%) |

All 19 GAEU-1 items are reported for completeness. Primary invariance analyses used the 15 SRL, motivation, and deep-learning indicators.

# Supplementary Table S3. Country-specific standardized CFA loadings and thresholds

| **Item** | **Factor** | **Chile loading** | **Ecuador loading** | **Chile thresholds t1-t4** | **Ecuador thresholds t1-t4** |
| --- | --- | --- | --- | --- | --- |
| GA2 | SRL | 0.746 | 0.699 | -3.021; -2.168; -1.224; 0.273 | -2.744; -1.707; -0.712; 0.472 |
| GA3 | SRL | 0.663 | 0.626 | -2.985; -2.343; -1.354; -0.146 | -3.046; -1.935; -0.884; 0.296 |
| GA4 | SRL | 0.506 | 0.601 | -2.592; -1.832; -0.827; 0.358 | -2.901; -1.995; -0.965; 0.289 |
| GA6 | SRL | 0.752 | 0.696 | -3.660; -2.721; -1.403; 0.610 | -3.650; -2.379; -1.073; 0.390 |
| GA13 | SRL | 0.756 | 0.693 | -2.835; -1.931; -0.640; 0.976 | -3.146; -1.784; -0.555; 0.875 |
| GA14 | SRL | 0.719 | 0.705 | -4.244; -2.551; -1.188; 0.717 | -3.698; -2.735; -1.026; 0.655 |
| GA7 | MOT | 0.657 | 0.491 | -1.666; -1.096; -0.214; 0.765 | -1.429; -0.760; 0.019; 0.879 |
| GA8 | MOT | 0.783 | 0.725 | -3.022; -2.303; -1.164; 0.450 | -2.716; -2.023; -0.813; 0.507 |
| GA9 | MOT | 0.840 | 0.811 | -4.450; -3.826; -2.163; -0.136 | -4.179; -3.008; -1.677; 0.204 |
| GA10 | MOT | 0.898 | 0.898 | -5.490; -4.576; -2.939; -0.693 | -5.550; -4.253; -2.424; -0.296 |
| GA11 | MOT | 0.855 | 0.851 | -4.813; -3.773; -2.133; -0.398 | -4.529; -3.287; -1.949; -0.171 |
| GA16 | DEEP | 0.817 | 0.674 | -3.389; -2.209; -1.083; 0.245 | -2.659; -1.764; -0.629; 0.602 |
| GA17 | DEEP | 0.773 | 0.686 | -2.858; -1.925; -0.533; 0.953 | -2.518; -1.423; -0.194; 1.157 |
| GA18 | DEEP | 0.797 | 0.744 | -3.785; -2.905; -1.624; 0.115 | -3.391; -2.346; -0.903; 0.592 |
| GA19 | DEEP | 0.643 | 0.565 | -1.900; -1.265; -0.112; 0.980 | -2.071; -1.203; -0.073; 1.092 |

# Supplementary Table S4. Complete differential item functioning results

| **Item** | **Factor** | **Uniform q** | **Non-uniform q** | **Total q** | **Delta R2 uniform** | **Delta R2 non-uniform** | **DIF classification** |
| --- | --- | --- | --- | --- | --- | --- | --- |
| GA16 | DEEP | 0.978 | 0.301 | 0.361 | 0.0000 | 0.0009 | No practical DIF |
| GA17 | DEEP | 0.551 | 0.779 | 0.655 | 0.0001 | 0.0001 | No practical DIF |
| GA18 | DEEP | 0.005 | 0.830 | 0.011 | 0.0036 | 0.0001 | No practical DIF |
| GA19 | DEEP | 0.007 | 0.779 | 0.016 | 0.0026 | 0.0001 | No practical DIF |
| GA10 | MOT | 0.387 | 0.997 | 0.655 | 0.0003 | 0.0000 | No practical DIF |
| GA11 | MOT | 0.354 | 0.830 | 0.585 | 0.0005 | 0.0000 | No practical DIF |
| GA7 | MOT | 0.387 | 0.002 | 0.002 | 0.0002 | 0.0040 | No practical DIF |
| GA8 | MOT | 0.287 | 0.779 | 0.458 | 0.0005 | 0.0001 | No practical DIF |
| GA9 | MOT | 0.296 | 0.830 | 0.522 | 0.0006 | 0.0000 | No practical DIF |
| GA13 | SRL | 0.027 | 0.117 | 0.011 | 0.0018 | 0.0017 | No practical DIF |
| GA14 | SRL | 0.287 | 0.779 | 0.406 | 0.0007 | 0.0002 | No practical DIF |
| GA2 | SRL | 0.007 | 0.779 | 0.016 | 0.0028 | 0.0001 | No practical DIF |
| GA3 | SRL | 0.000 | 0.301 | 0.000 | 0.0092 | 0.0011 | No practical DIF |
| GA4 | SRL | 0.018 | 0.779 | 0.026 | 0.0024 | 0.0003 | No practical DIF |
| GA6 | SRL | 0.027 | 0.426 | 0.026 | 0.0021 | 0.0007 | No practical DIF |

Classification used the study rule: BH-FDR q < .05 AND Delta McFadden R2 >= .02 for non-negligible DIF. DIF served as complementary localization evidence rather than a mandatory partial-invariance gate; results for the four-item deep-learning factor should be interpreted cautiously. For GA3, the maximum total Delta R2 of .0103 equals, to rounding, .0092 uniform plus .0011 non-uniform components; the manuscript now states this decomposition explicitly.

# Supplementary Table S5. Partial-invariance decision log

| **Stage** | **Item** | **Parameter** | **Local/score-test evidence** | **DIF evidence** | **Content rationale** | **Decision** | **Effect on fit** |
| --- | --- | --- | --- | --- | --- | --- | --- |
| All stages | — | — | Threshold, loading, and scalar-equivalent constraints satisfied the finalized incremental guide. | No item met the practical DIF criterion. | No parameter-specific release rationale required. | No parameters freed | Not applicable |

No partial-invariance decision was required because equality constraints were retained through the strong/scalar stage. Residual variances were not constrained, so strict invariance was not tested. The planned release procedure was therefore unused.

# Supplementary Table S6. Final invariant anchors and released parameters

| **Factor** | **Item** | **Threshold status** | **Loading status** | **Intercept-related status** | **Untouched anchor?** |
| --- | --- | --- | --- | --- | --- |
| SRL | GA2 | Invariant | Invariant | Invariant | Yes |
| SRL | GA3 | Invariant | Invariant | Invariant | Yes |
| SRL | GA4 | Invariant | Invariant | Invariant | Yes |
| SRL | GA6 | Invariant | Invariant | Invariant | Yes |
| SRL | GA13 | Invariant | Invariant | Invariant | Yes |
| SRL | GA14 | Invariant | Invariant | Invariant | Yes |
| MOT | GA7 | Invariant | Invariant | Invariant | Yes |
| MOT | GA8 | Invariant | Invariant | Invariant | Yes |
| MOT | GA9 | Invariant | Invariant | Invariant | Yes |
| MOT | GA10 | Invariant | Invariant | Invariant | Yes |
| MOT | GA11 | Invariant | Invariant | Invariant | Yes |
| DEEP | GA16 | Invariant | Invariant | Invariant | Yes |
| DEEP | GA17 | Invariant | Invariant | Invariant | Yes |
| DEEP | GA18 | Invariant | Invariant | Invariant | Yes |
| DEEP | GA19 | Invariant | Invariant | Invariant | Yes |

All 15 primary indicators remained invariant anchors: SRL = 6, motivation = 5, deep learning = 4.

# Supplementary Table S7. Sensitivity analysis excluding zero-variance response patterns using the same final invariance/partial specification

| **Result** | **Primary sample** | **Sensitivity sample** | **Difference / conclusion** |
| --- | --- | --- | --- |
| N | 1,316 | 1,307 | Nine zero-variance response patterns excluded |
| Configural CFI | 0.950 | 0.949 | Essentially unchanged |
| Threshold Delta CFI | -0.001 | -0.001 | Same incremental-fit conclusion |
| Loading Delta CFI | +0.005 | +0.005 | Same incremental-fit conclusion |
| SRL latent Cohen d | -0.096 | -0.099 | Conclusion unchanged |
| Motivation latent Cohen d | -0.225 | -0.228 | Conclusion unchanged |
| Deep learning latent Cohen d | -0.284 | -0.292 | Conclusion unchanged |

# Supplementary Table S8. Data-informed local-dependence sensitivity using the same final invariance/partial specification

| **Model** | **Residual associations** | **CFI** | **TLI** | **RMSEA** | **SRMR** | **Invariance conclusion changed?** | **Latent effect changed?** |
| --- | --- | --- | --- | --- | --- | --- | --- |
| Primary | None | 0.950 | 0.939 | 0.089 | 0.059 | — | Reference |
| Sensitivity | GA2 ~~ GA3; GA7 ~~ GA8 | 0.966 | 0.958 | 0.074 | 0.052 | No | SRL d = -0.092; MOT d = -0.233; DEEP d = -0.284 |

The data-informed local-dependence model improved global fit while preserving the strong/scalar-level invariance result and essentially unchanged latent contrasts. The residual links were not theory-prespecified: GA2-GA3 and GA7-GA8 were selected after preliminary diagnostics. Their content is nevertheless interpretable because the first pair concerns study-setting preparation and the second pair motivational self-talk. The model remains a sensitivity analysis rather than the primary specification.

# Supplementary Table S9. Latent factor correlations from the final strong/scalar-level ordinal model

| Country | Factor pair | r | 95% CI |
| --- | --- | --- | --- |
| Chile | GAEU-1 SRL - Conscious motivation | .758 | [.714, .802] |
| Chile | GAEU-1 SRL - Deep learning | .836 | [.799, .872] |
| Chile | Conscious motivation - Deep learning | .675 | [.615, .734] |
| Ecuador | GAEU-1 SRL - Conscious motivation | .739 | [.693, .785] |
| Ecuador | GAEU-1 SRL - Deep learning | .763 | [.711, .814] |
| Ecuador | Conscious motivation - Deep learning | .579 | [.514, .644] |

Note. Values are standardized latent-factor correlations from the final strong/scalar-level model and were freely estimated by country; residual variances were not constrained across groups. The Chilean GAEU-1 SRL-deep-learning correlation (r = .836) indicates substantial overlap. Supplementary Table S11 adds Wald contrasts against r = 1 and Fornell-Larcker diagnostics; these show that the factors are not statistically identical but that discriminant separation is incomplete for pairs involving the GAEU-1 SRL factor.

**Supplementary Table S10. Model-parameter counts and robust scaled nested chi-square difference tests**

| **Model** | **Constraints** | **Model parameters** | **Scaled chi-square (df)** | **Comparison** | **Scaled Delta chi-square (Delta df)** | **p** |
| --- | --- | --- | --- | --- | --- | --- |
| M1 | Configural | 156 | 1084.88 (174) | — | — | — |
| M2 | Thresholds | 186 | 1136.11 (204) | M2 vs M1 | 54.47 (30) | .004 |
| M3 | Thresholds + loadings | 189 | 1055.24 (216) | M3 vs M2 | 27.06 (12) | .008 |
| M4 | Thresholds + loadings + intercept-related | 177 | 1072.27 (228) | M4 vs M3 | 54.76 (12) | < .001 |

Note. “Model parameters” is the number reported by lavaan for each WLSMV model under the Wu-Estabrook categorical identification. Counts need not decline monotonically across stages because threshold/intercept parameterization changes with the imposed equality constraints. Robust scaled difference tests use the Satorra (2000) method reported by semTools::compareFit. The statistically significant exact tests are interpreted alongside the very small changes in CFI/RMSEA, not as a sole gate for invariance.

**Supplementary Table S11. Post hoc discriminant-validity diagnostics**

| **Country** | **Factor pair** | **r** | **SE** | **Wald z: H0 r=1** | **p** | **sqrt(AVE) pair** | **Fornell-Larcker** | **Polychoric HTMT** |
| --- | --- | --- | --- | --- | --- | --- | --- | --- |
| Chile | SRL-MOT | .758 | .0224 | -10.82 | < .001 | .696 / .811 | Not met | 0.749 |
| Chile | SRL-DEEP | .836 | .0185 | -8.88 | < .001 | .696 / .761 | Not met | 0.836 |
| Chile | MOT-DEEP | .675 | .0303 | -10.74 | < .001 | .811 / .761 | Met | 0.695 |
| Ecuador | SRL-MOT | .739 | .0236 | -11.06 | < .001 | .671 / .769 | Not met | 0.767 |
| Ecuador | SRL-DEEP | .763 | .0262 | -9.06 | < .001 | .671 / .671 | Not met | 0.755 |
| Ecuador | MOT-DEEP | .579 | .0332 | -12.70 | < .001 | .769 / .671 | Met | 0.609 |

Note. Wald z = (r - 1)/SE from the freely estimated standardized latent correlation. AVE was calculated as the mean squared completely standardized loading within each factor. The Fornell-Larcker diagnostic is met when the square root of AVE for both factors exceeds their absolute correlation. Polychoric HTMT was calculated directly from the ordered-item polychoric correlation matrix; .85 is shown only as a conventional descriptive benchmark. All factor correlations are statistically below unity and all HTMT values are below .85, whereas Fornell-Larcker indicates incomplete discriminant separation for pairs involving the GAEU-1 SRL factor. These are post hoc diagnostics conducted after the primary analysis plan had been finalized, not preregistered confirmatory tests.

**Supplementary Table S12. Post hoc sensitivity checks for statistical sensitivity and practical-DIF threshold choice**

| **Panel / analysis** | **Specification** | **Result** | **Interpretation** |
| --- | --- | --- | --- |
| A. RMSEA power | Chile CFA: df=87, n=631; H0 RMSEA=.05 vs H1=.08 | Power > .999 | High sensitivity to appreciable absolute misfit |
| A. RMSEA power | Ecuador CFA: df=87, n=685; H0 RMSEA=.05 vs H1=.08 | Power > .999 | High sensitivity to appreciable absolute misfit |
| A. RMSEA power | M1-M4: df=174-228, N=1,316; H0 RMSEA=.05 vs H1=.08 | Power > .999 for each stage | Low power is not a plausible explanation for elevated RMSEA |
| B. DIF threshold | .010 practical Delta McFadden R2 + q<.05 | 1 item (GA3) | Conclusion becomes more conservative at .010 |
| B. DIF threshold | .015 practical Delta McFadden R2 + q<.05 | 0 items | No item reaches threshold |
| B. DIF threshold | .020 practical Delta McFadden R2 + q<.05 | 0 items | Prespecified classification used in main analysis |

Note. RMSEA power follows the noncentral chi-square framework of MacCallum, Browne, and Sugawara (1996), using alpha = .05. This is a sensitivity analysis for global model misfit, not a retrospective “observed power” transformation of the obtained p values. The DIF panel does not claim a formal item-level power calculation; it shows directly how the substantive classification changes as the practical-effect threshold is varied.

**Supplementary Table S13. Post hoc country-specific residual and modification-index diagnostics**

| **Country** | **Largest residual pair** | **Residual r** | **Std. residual z** | **Largest covariance MI pair** | **MI** | **EPC** |
| --- | --- | --- | --- | --- | --- | --- |
| Chile | GA7-GA8 | .174 | 8.46 | GA7 ~~ GA8 | 91.85 | .596 |
| Ecuador | GA7-GA8 | .201 | 8.64 | GA7 ~~ GA8 | 79.38 | .461 |

Note. Diagnostics come from the retained country-specific three-factor ordinal CFA models and were conducted post hoc after the primary analysis plan had been finalized. GA2-GA3 was the second-largest residual-covariance modification-index pair in both countries (Chile MI=35.78; Ecuador MI=24.78). No residual covariance was added to the primary model from these post hoc diagnostics.

**Supplementary Table S14. Post hoc one-factor versus retained three-factor ordinal CFA comparison**

| **Country** | **Structure** | **Scaled chi-square (df)** | **CFI** | **TLI** | **RMSEA** | **SRMR** | **Delta CFI / Delta RMSEA vs 3F** |
| --- | --- | --- | --- | --- | --- | --- | --- |
| Chile | Retained 3-factor | 575.36 (87) | .947 | .937 | .094 | .059 | Reference |
| Chile | One-factor | 1129.73 (90) | .888 | .869 | .135 | .088 | -.059 / +.041 |
| Ecuador | Retained 3-factor | 512.95 (87) | .952 | .942 | .085 | .059 | Reference |
| Ecuador | One-factor | 1128.58 (90) | .883 | .863 | .130 | .089 | -.069 / +.045 |

Note. The one-factor model was added post hoc as a structural sensitivity analysis. The comparison is descriptive and is not an adjacent invariance-constraint test. Poorer one-factor fit argues against simple unidimensionality but does not establish the retained three-factor model as uniquely optimal.

**Supplementary Table S15. Private-university-only ordinal invariance sensitivity analysis**

| **Model** | **CFI** | **TLI** | **RMSEA** | **SRMR** | **Delta CFI** | **Delta RMSEA** | Incremental-fit result |
| --- | --- | --- | --- | --- | --- | --- | --- |
| M1 | .949 | .939 | .089 | .062 | - | - | Reference |
| M2 | .948 | .947 | .083 | .062 | -.001 | -.006 | Meets guide |
| M3 | .953 | .955 | .077 | .062 | +.005 | -.006 | Meets guide |
| M4 | .954 | .957 | .074 | .062 | <+.001 | -.002 | Meets guide |

Note. Restricted sample N=1,037 (Chile n=363; Ecuador n=674). M1=configural; M2=threshold equality; M3=threshold + loading equality; M4=threshold + loading + intercept-related equality. Standardized Ecuador-minus-Chile latent mean estimates from M4 were SRL=-.071, motivation=-.278, and deep learning=-.273. The restriction is a composition-sensitivity check, not an institution-matched analysis; women comprised 35.8% of the Chilean private-only sample and 66.0% of the Ecuadorian private-only sample.

# Supplementary reproducibility note

The primary archived workflow was run in R 4.6.0 on macOS Tahoe 26.6 (aarch64-apple-darwin23) with lavaan 0.7-2, semTools 0.5-8, lordif 0.4.2, mirt 1.47, psych 2.6.5, and ggplot2 4.0.3. The post hoc Revision-1 rerun used the same R/lavaan environment with semTools 0.5-9; its session information is included in Supplementary File 2. Observed and latent comparisons use commensurate Cohen-d-type standardized metrics. Formal inference relies on model-estimated latent mean differences and Holm-adjusted p values; the sample contrasts are descriptive rather than estimates of isolated country effects.

Supplementary File 2. Reproducibility materials

Supplementary File 2 contains the analysis protocol, R scripts for the reported analyses, configuration files, package/session information, model syntax, diagnostics, machine-readable primary and sensitivity output tables, figure source output, and the complete verified outputs from the post hoc diagnostic and robustness analyses (country-specific residual diagnostics, one-factor comparison, HTMT, and private-university-only sensitivity). Respondent-level raw data are not duplicated; the source data are publicly available from Mendeley Data (DOI: 10.17632/7vgyndhb87.5).
